# Confocal Microscopy for Diagnosis and Management of Cutaneous Malignancies: Clinical Impacts and Innovation

**DOI:** 10.3390/diagnostics13050854

**Published:** 2023-02-23

**Authors:** Mehmet Fatih Atak, Banu Farabi, Cristian Navarrete-Dechent, Gennady Rubinstein, Milind Rajadhyaksha, Manu Jain

**Affiliations:** 1 Department of Dermatology, New York Medical College, Metropolitan Hospital, New York, NY 10029, USA; 2 Department of Dermatology, Escuela de Medicina, Pontificia Universidad Catolica de Chile, Santiago 8331150, Chile; 3 Dermatology and Laser Centre, Los Angeles, CA 91604, USA; 4 Dermatology Service, Department of Medicine, Memorial Sloan Kettering Cancer Center, New York, NY 10065, USA; 5 Dermatology Service, Department of Medicine, Weill Cornell Medicine, New York, NY 10021, USA

**Keywords:** confocal microscopy, reflectance confocal microscopy, in vivo confocal microscopy, ex vivo confocal microscopy, innovations and advances in confocal microscopy in cutaneous oncology, clinical impacts in confocal microscopy in cutaneous oncology

## Abstract

Cutaneous malignancies are common malignancies worldwide, with rising incidence. Most skin cancers, including melanoma, can be cured if diagnosed correctly at an early stage. Thus, millions of biopsies are performed annually, posing a major economic burden. Non-invasive skin imaging techniques can aid in early diagnosis and save unnecessary benign biopsies. In this review article, we will discuss in vivo and ex vivo confocal microscopy (CM) techniques that are currently being utilized in dermatology clinics for skin cancer diagnosis. We will discuss their current applications and clinical impact. Additionally, we will provide a comprehensive review of the advances in the field of CM, including multi-modal approaches, the integration of fluorescent targeted dyes, and the role of artificial intelligence for improved diagnosis and management.

## 1. Introduction

Skin cancer is the most common cancer worldwide, with rising incidence [1]. The most common skin cancer is basal cell carcinoma (BCC), followed by squamous cell carcinoma (SCC), and melanoma [1,2,3]. Fortunately, when detected at an early stage, most skin cancers can be cured, including the deadliest cancer, melanoma [2,4]. Current diagnostic methods for detection of skin cancer are visual (naked eye) examination and dermoscopy. Although dermoscopy has improved the sensitivity for diagnosing skin cancers from 70.6% to 84.6% compared with visual examination alone, specificity of dermoscopy remains low (86%). This has resulted in a higher number needed to excise (NNE) of 5.23 with dermoscopy compared with visual evaluation alone (4.77), which leads to unnecessary biopsies of benign tissue [5].

Lower specificity of dermoscopic diagnosis is related to a lack of cellular resolution [6,7]. Thus, a biopsy is often performed for histopathological confirmation [8]. Biopsy is an invasive procedure that can be associated with complications such as bleeding, infection, delayed healing, and scars. Furthermore, a biopsy is a terminal procedure and does not allow to follow-up changes in a lesion over time. Although histopathology is the gold-standard, it cannot give an immediate bedside diagnosis due to time-consuming tissue processing, which may delay management and increase patients’ anxiety. Moreover, the majority of the lesions biopsied to rule-out cancers are diagnosed as benign. Thus, the overall diagnosis and management of skin cancer poses a heavy cost burden to public health and society at large [8,9,10].

To improve specificity and to detect skin cancers at earlier stages, non-invasive optical imaging techniques were developed in recent decades; confocal microscopy (CM) is one such technique. There are two types of CM: reflectance CM (RCM) and ex vivo CM (EVCM). Use of RCM can image skin lesions at a “quasi-histologic” level, in vivo, without need to perform a biopsy. Imaging relies solely on the reflectance contrast from various tissue components of the skin and does not use any exogenous contrast agent or dye (i.e., it is ‘label free’) [11]. The RCM device has acquired current procedural terminology (CPT) billing codes in the US and is primarily used to diagnose neoplastic and non-neoplastic skin lesions [12,13]. Lesions that are diagnosed benign on RCM are spared a biopsy (reducing unnecessary biopsies), while skin cancers, depending on their stage (early or late), proceed directly to treatment (surgical or nonsurgical). The use of RCM has also increased the accuracy of the non-invasive diagnosis of melanocytic [14] and non-melanocytic [15] skin cancers. For instance, the NNE for diagnosing melanoma has dropped to 3.0 with RCM (compared with 5.3 for dermoscopy alone) [7]. Moreover, RCM can be utilized for the surveillance of the recurrence of melanoma [16] or BCC [17], as well as the selection of appropriate treatment modalities for skin malignancies [18,19]. Importantly, reduced biopsy rates and the early detection of skin cancer have been shown to reduce the financial burden of skin cancer detection [10]. Although RCM has been shown to be valuable for the diagnosis and management of cutaneous tumors, there are certain limitations with the current commercial devices. These limitations are mostly due to the inherent nature of this technology, such as en-face visualization of the tissue, limited depth of imaging (~200 µm), small field of view (FOV) images (when using the handheld RCM device), grayscale images, and a lack of cellular specificity (i.e., the inability to differentiate dendritic melanocytes from Langerhans cells). Largely due to these difficulties with image interpretation and high cost, the adoption of RCM remains limited worldwide, despite its usefulness [20]. 

Unlike the RCM device, the EVCM device is used to image freshly excised tissues. While RCM imaging does not require dye application to the skin, EVCM imaging tissues are stained with a fluorescent nuclear dye. Thus, EVCM can be used to image tissues both in reflectance and fluorescence modes. The signals captured in these two modes are combined and can be digitally colored as purple-pink images (reflectance signal from collagen and cytoplasm in pink and fluorescent nuclear signal in purple), simulating hematoxylin and eosin (H&E)-stained histopathology [21]. This device is currently being integrated into Mohs surgery for the assessment of tumor margins [22]; however, it also can be used to evaluate skin lesions rapidly and as a potential adjunct tool to conventional histopathology evaluations. In particular, it could also be a valuable tool in resource-poor countries and remote areas where a histopathology laboratory set-up is not available. 

In this review article, we will summarize the current evidence of both RCM and ECVM for their clinical impact on the diagnosis and management of skin cancers. Since the inception of CM, several innovations have occurred to improve the diagnostic accuracy of these devices and make them more user-friendly and widely available. These include: (1) building a combined RCM and optical coherence tomography (OCT) device (i.e., a multimodal approach), (2) the use of targeted molecular probes, (3) building cheaper and portable microscopes, and (4) integrating artificial intelligence (AI) algorithms to aid novices with image interpretation and diagnosis. 

## 2. Current Application of Confocal Microscopy (CM)

### 2.1. Current Applications of Reflectance CM (RCM)

Features of skin cancers identifiable on RCM have been described in the literature [23,24,25]. The most common features of skin malignancies seen in RCM and their histopathologic correlates are summarized in Supplementary Table S1.

#### 2.1.1. Skin Cancer Diagnosis and Management with RCM 

**Figure 1:**
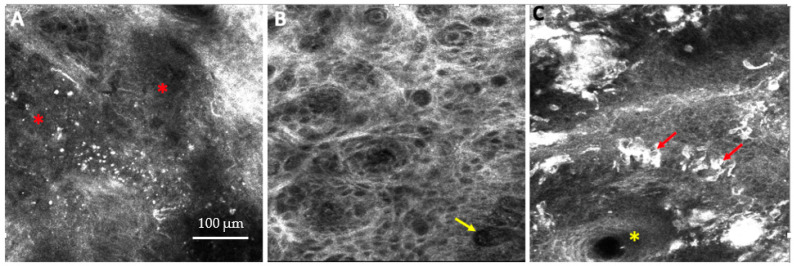
(**A**–**C**) Reflectance confocal microscopy (RCM) images of (**A**) basal cell carcinoma (BCC) showing tumor nodules (red asterisk), (**B**) squamous cell carcinoma showing an atypical honeycomb pattern at the spinosum layer with prominent vessels (yellow arrow), and (**C**) melanoma showing clusters of atypical melanocytes (red arrows) in the epidermis and around a hair follicle (yellow asterisk). Field of view: (**A**–**C**) = 500 × 500 µm. Image A courtesy of Ms. Rozina Zeidan, Clinical Research Specialist, Memorial Sloan Kettering Cancer Center.

Providing images at cellular resolution at the bedside in real time, RCM has been established as a valuable diagnostic tool for the detection of skin cancers, including melanocytic and keratinocyte cancers [26] (Figure 1A–C and Figure S1A).

##### Melanoma Diagnosis

Melanin has the highest refractive index, and therefore, appears bright on RCM, which makes it easy to visualize melanocytic lesions, including melanoma [26]. Dinnes et al. performed a test of diagnostic accuracy and showed that, at a fixed sensitivity of 90%, RCM’s specificity was 82% for lesions clinically suspicious for melanoma and 86% for clinically equivocal lesions, compared with 42% and 49% on dermoscopy alone, respectively [14]. A subsequent meta-analysis conducted by Pezzini et al. reported similar results for the detection of melanoma via RCM, with a sensitivity of 92% (95% CI: 0.91–0.93) and a specificity of 70% (95% confidence interval [CI], 0.69–0.71). They also demonstrated that the use of RCM improves the accuracy of diagnosing melanoma compared with dermoscopy alone, achieving a sensitivity of 96% (95% CI, 0.93–0.98; I^2^ = 75%) vs. 90% (95% CI, 0.86–0.93, I^2^ = 0%), and specificity of 56% (95% CI, 0.52–0.60; I^2^ = 97%) vs. 38% (95% CI, 0.34–0.42; I^2^ = 98%), respectively [27]. In addition to its use in classic pigmented melanomas, RCM is also valuable for the diagnosis of amelanotic/hypomelanotic melanomas. In such cases, RCM has demonstrated a higher sensitivity of 67% (95% CI, 0.51–0.81) compared with 61% [95% CI, 0.37–0.81] for dermoscopy alone, with a similar specificity for both techniques (89% for RCM vs. 90% for dermoscopy alone) [28]. All of the aforementioned studies demonstrated that an increased diagnostic accuracy with RCM not only led to earlier detection of melanoma but has also reduced the rate of unnecessary benign excisions. To this end, a recent study by Pellacani et al. demonstrated that the use of RCM combined with dermoscopy reduces the NNE by 43.3% (from 5.3 to 3.0) in lesions suspected to be melanoma, as compared with the use of dermoscopy alone [7]. Furthermore, a cost–benefit analysis conducted in Italy showed that the routine use of RCM saved approximately EUR 260,000 per year per million inhabitants [10].

In addition to diagnosing melanoma in a new lesion, RCM shows promising results in detecting early melanoma changes in an existing lesion when used for a non-invasive imaging follow-up. Lesion monitoring performed with digital dermoscopy has a sensitivity of 71.4% and a specificity of 63.4%, which could be increased to a 100% sensitivity with the addition of RCM [29].

In particular, RCM has been shown to be most useful for the diagnosis of melanomas located on the head and neck regions, on chronically sun-damaged skin, and for lesions with regression on dermoscopy. Lesions arising on the head and neck tend to be challenging due to the frequent presence of multiple freckles; solar lentigines; and flat, pigmented lesions that can mimic melanoma. Additionally, since it is a highly cosmetic and functional site, clinicians tend to avoid performing biopsies. RCM emerges as a valuable tool for the evaluation of lesions on these sites. Borsari et al. demonstrated that a higher diagnostic accuracy achieved through RCM significantly correlated with lesions located on sun damaged skin (Spearman analysis ρ = 0.149; *p* < 0.001) and lesions with dermoscopically observed regression (ρ = 0.096; *p* = 0.001) [30]. Lentigo maligna (LM) and its invasive counterpart LM melanoma (LMM) are the most common melanomas seen on chronically sun-damaged skin [31]. LMMs grow gradually during their horizontal (intraepidermal) growth phase and have a low mortality potential; however, they often mimic non-melanocytic neoplasms (pigmented actinic keratosis, seborrheic keratosis, solar lentigo, etc.), requiring biopsy for confirmation. To diagnose LM on the face within pigmented and non-pigmented equivocal lesions, diagnosticians have developed a scoring system based on RCM features; an LM score consists of two major features (nonedged papillae and pagetoid cells round and >20 mm in diameter, each scored two points) and three minor features (three or more atypical cells at the junction in five images, the follicular localization of pagetoid cells and/or atypical junctional cells, and nucleated cells within the papilla). An LM score of ≥2 on RCM has a sensitivity of 85% and specificity of 76% for the diagnosis of LM [32]. RCM may also be useful to discriminate LM from metal-induced cutaneous hyperpigmentation (e.g., tattoo, chrysiasis, argyriasis) [33].

RCM has a limited role in the diagnosis of nodular and acral melanomas. Although nodular melanomas can be visualized when located in the superficial dermis, deeper melanomas can be missed due to RCM imaging’s limited depth of penetration (~250 µm) [20]. Likewise, acral sites have a thick stratum corneum which may hinder the visualization of melanoma due to high reflectance from the thick keratin layer [34]. Awareness of these limitations is crucial to prevent false negative diagnoses and subsequently devastating consequences.

##### Margin Assessment and Surveillance of Melanoma

LMs/LMMs are large ill-defined lesions with subclinical extensions that are challenging to detect visually and even with dermoscopy [35]. An inaccurate margin assessment often leads to incomplete excision of these lesions, increasing the risk of recurrence and additional surgery [36]. RCM is a highly valuable tool for the presurgical delineation of LM/LMM margins [37]. In these cases, the handheld RCM device [36] plays an important role, as it can be moved freely over the skin to map the lesion’s extension. This device has shown a high sensitivity of 90% and a specificity of 86% for detecting these lesion types [37]. Guitera et al. reported that the use of RCM can identify the subclinical extension of LM 5 mm beyond the dermoscopically detected margin in 59% of patients [18]. The handheld device can also be used to guide biopsy acquisition in the most suspicious areas to assess invasion [38]. The device may also be used to select an appropriate treatment modality (i.e., surgical vs. non-surgical [radiotherapy or topical immunotherapy]) for treatment of these cancers [18]. This study also showed that, with the use of RCM, disease management strategies were changed in 73% of patients [18]. As compared with dermoscopy alone, the use of perioperative RCM was found to aide LM mapping during slow Mohs surgery and reduce the mean number of layers required for tumor clearance (from 1.54 layers to 1.29) and the mean time to repair (from 27 days to 14.6) (*p* < 0.05) [39]. 

Not only is RCM useful for margin assessments, but it can also be used to detect LM/LMM recurrence in scar tissue after surgical excision [16]. It is also useful for monitoring the response of unresectable melanomas to non-surgical therapies such as topical imiquimod, immunotherapy, and radiotherapy [40,41].

##### Basal Cell Carcinoma (BCC) Diagnosis and Management

RCM also plays an important role in the diagnosis and management of BCC, the most common skin cancer. There are several benign clinical mimics of BCC, most of which are present on the face. If diagnosed accurately in vivo, benign lesions can be spared biopsies, while BCCs can be triaged for a surgical or non-surgical approach (e.g., photodynamic therapy (PDT) or imiquimod for superficial and nodular lesions). A meta-analysis of 15 studies showed that RCM has a high pooled sensitivity and specificity of 92% (95% CI, 0.87–0.95; I^2^ = 85.27%) and 93% (95% CI, 0.85–0.97; I^2^ = 94.61%), respectively, for the diagnosis of BCC [42]. However, these studies did not specify the level of clinical difficulty in diagnosing these lesions, thus lacking evidence of RCM’s utility for clinically challenging lesions. A randomized controlled multicenter trial compared RCM with punch biopsy for the diagnosis of BCC and demonstrated that RCM has a lower specificity (59.1% vs. 100.0%; *p* < 0.001), with a comparable sensitivity of 99.0% (*p* = 1.0) [43]. Additionally, the investigators demonstrated that RCM’s sensitivity for the diagnosis of non-superficial BCC was not significantly different from that of punch biopsy (88.9% for RCM vs. 91.0% for biopsy; *p* = 0.724); furthermore, patient satisfaction was highly comparable for these two methods [43]. RCM is also valuable for the diagnosis and sub-typing of BCC and guidance of management (surgical vs. non-surgical), saving time, reducing cost, and improving comfort for patients [19,44]. 

The accuracy of diagnosing BCC on RCM has been compared with dermoscopy alone, especially for equivocal pink or lightly pigmented BCCs. Witkowski et al. demonstrated RCM’s specificity to be slightly higher (93.8%) compared with dermoscopy alone (92.4%), though both had the same sensitivity (85.1%) [45]. A recent article by Dinnes et al. reported that, compared with dermoscopy, RCM demonstrated a higher sensitivity (94% vs. 85%), but a lower specificity (85% vs. 92%) [15]. 

Superficial BCC can be treated non-surgically when diagnosed early. This includes treatments such as topical therapies (e.g., 5% imiquimod, 5% fluorouracil) and destructive approaches (e.g., curettage, electrocautery, cryotherapy, laser ablation) [46]. Similar to its application for LM, RCM is also useful for monitoring response to non-invasive treatment of BCC. Guida et al. showed a pooled sensitivity of 100% and specificity of 72.5% for detecting persistent BCC after PDT or treatment with vismodegib [17].

RCM has been found to be effective in detecting residual BCC on clinically negative biopsy sites prior to Mohs surgery. Navarrete-Dechent et al. have shown a sensitivity of 92.8% and a specificity of 68.4% for the detection of residual BCC with RCM [47]. RCM has also been used intra-operatively to detect residual nonmelanoma skin cancers in lateral and deeper margins during Mohs surgery. In these cases, the use of RCM was able to detect residual tumors in 88% of lesions [48]. Similar results of 88.5% sensitivity and 91.7% specificity were reported with the use of RCM for the detection of BCC in wound margins [49]. Finally, RCM has been used to guide treatment with ablative lasers (such as CO_2_ lasers) [19]; with such use, a total of 22% of cases required additional laser passes after real-time RCM evaluation of the treated site.

##### Squamous Cell Carcinoma (SCC) Diagnosis and Management

Similar to its use in the diagnosis of melanoma and BCC, RCM has been assessed for its efficacy for the diagnosis of SCC in equivocal lesions. A recent database meta-analysis demonstrated a sensitivity of 74–77% and a high specificity of 92–98% for the detection of SCC [15]. Another study showed a similar sensitivity of 74% (95% CI, 58–86%) and a specificity of 92% (95% CI, 88–95%) when the images were read by an experienced reader [50]. However, novices performed with a low sensitivity of 41%, but surprisingly, a higher specificity of 97% [50]. In contrast to its demonstrated value for diagnosis and management of melanoma and BCC, more studies are needed to confirm the role of RCM for SCC.

### 2.2. Current Applications of Ex Vivo Confocal Microscopy (EVCM) in Dermatology 

**Figure 2:**
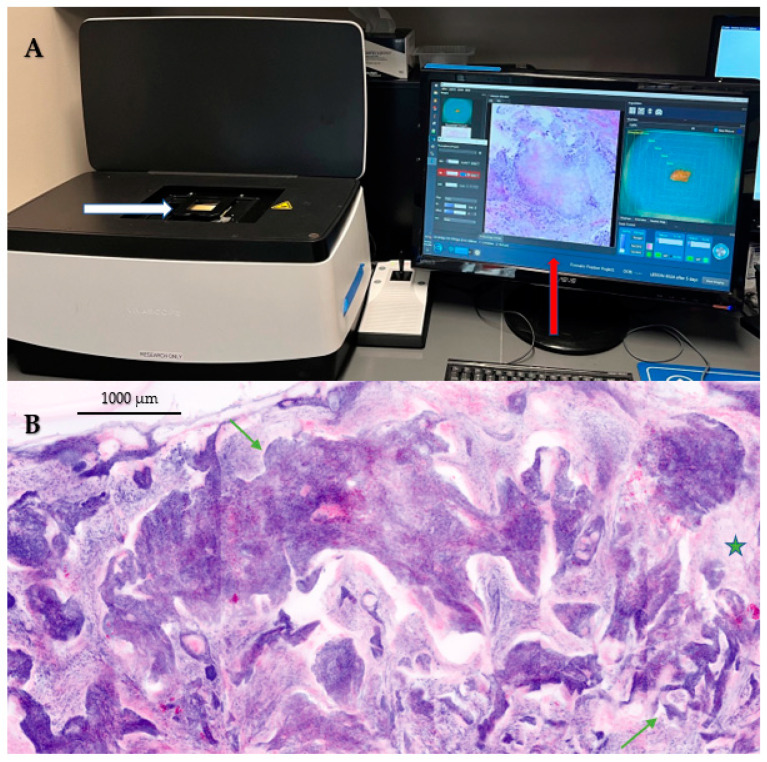
(**A**) An ex vivo confocal microscopy (EVCM) device with an attached computer screen for visualization of images in real-time. A fresh tissue from skin is mounted on a glass slide (white arrow) for imaging on the device and its digital hematoxylin and eosin (H&E) image (red arrow) is visible on the screen. (**B**) An EVCM image of a basal cell carcinoma in DHE mode showing nodular and infiltrative components (green arrows). Tumor nodules and cords appear purple due to fluorescent signals from nuclei, while stroma (green five-pointed star) appears pink due to reflectance signal, simulating H&E staining. Image A courtesy of Dr. Julia Kahn, Medical Graduate Student, Memorial Sloan Kettering Cancer Center.

Using EVCM equipment (Figure 2A), physicians and expert readers have defined features of various skin lesions, including benign and malignant neoplasms (Figure 2B) and inflammatory lesions [51]. EVCM’s major application is for the assessment of tumor margins during Mohs surgery. The device has shown a two-third reduction in time when compared with the requirements to process a frozen section (*p* < 0.001) [52]. 

#### 2.2.1. EVCM for Diagnosis and Management of Melanoma 

EVCM has been used to assess LM and LMM in surgical margins of 42 cutaneous and two mucosal LM/LMMs. The results for the diagnosis of cutaneous LM/LMM via EVCM were compared with those of in vivo RCM imaging [53]. The authors found that EVCM had a 95.5% rate of correct identification of tumor margins for both LM and LMM. This was comparable with the rate of 97.6% with cutaneous LM/LMM. Furthermore, EVCM demonstrated an ability to measure LM/LMM thickness in fresh tissues, which had a high correlation with the depth of tissue sections on histopathology; the mean difference was 0.09 ± 0.30 mm and 0.19 ± 0.35 mm on EVCM and histopathology. This study highlights the role of EVCM for perioperative decisions on safety margins for the excisions of LM/LMM in the future, potentially reducing time, cost, and the redundancy of processes [54].

#### 2.2.2. EVCM for the Intra-Operative Margin Assessment of Keratinocyte Carcinomas

The use of EVCM has shown high sensitivity (79.8%) and specificity (95.8%) with a 95.7% negative predictive value for the detection of BCC in surgical margins during Mohs surgery [55]. Moreover, EVCM can be used to subtype residual BCCs reliably with a high diagnostic accuracy (90% for superficial BCC, 83% for nodular BCC, and 86% for infiltrative BCC) [56]. EVCM is also a useful tool to evaluate the surgical margin assessment of cutaneous SCC [22,57]. Horn et al. demonstrated that EVCM has an overall high sensitivity of 95% and specificity of 96.25% for the diagnosis of SCC in freshly excised skin lesions [58].

## 3. Advances in the Field of Confocal Microscopy (CM)

### 3.1. Enlarging the Field of View (FOV) of RCM Images 

Although the arm-mounted RCM device can acquire images from a lesion measuring ≤ 8 × 8 mm, the FOV is smaller with the handheld device. To overcome this limitation, a video-mosaicking approach had been developed where “movies”, i.e., live videos, are stitched together to provide a larger FOV. However, these videos often have motion-related artifacts due to rapid movements during image acquisition, thus affecting image quality. To reduce these artifacts, several AI algorithms have been built [59].

### 3.2. Multimodal Imaging

Various optical imaging devices have been combined with CM to overcome the limitations of CM, especially an en-face view of images, limited imaging depth, and a lack of cellular specificity. Such devices include RCM-OCT and RCM-multiphoton microscopy (MPM). Below, we describe some of these devices and their potential clinical applications. 

#### 3.2.1. RCM-Optical Coherence Tomography (OCT) Device 

Recently, a combined RCM-OCT probe has been produced. OCT is another non-invasive optical imaging technique that has similar principles to ultrasound (US) imaging. While US imaging relies on the detection of signals generated by acoustic waves, OCT measures echo delays and the intensity of back-reflected infrared/near-infrared light [60]. Although the RCM device retrieves only a reflectance signal, the combination of OCT adds birefringence information from examined tissue. In RCM-OCT, both devices exist within a single handheld imaging probe and RCM and OCT images are co-registered [61] (Supplemental Figure S1B). While RCM provides high-resolution cellular-level information, OCT provides increased depth of imaging in a vertical mode, up to 1 mm (similar to histopathology) [61,62]. 

**Figure 3:**
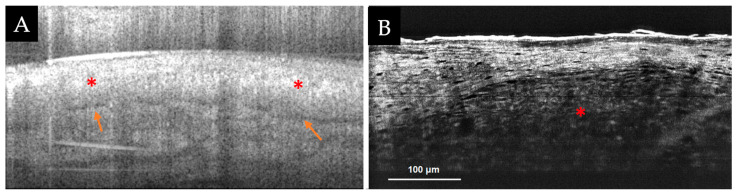
(**A**,**B**) Optical coherence tomography (OCT) images of tumor nodules (red asterisks) with clefting (orange arrows) from the BCC shown in Figure 1A acquired with (**A**, at left) an RCM-OCT probe, and (**B**, at right) high-resolution OCT device. Field of view: (**A**) = 2 × 1 mm; (**B**) = 500 × 400 µm. Images (**A**,**B**) courtesy of Ms. Rozina Zeidan, Clinical Research Specialist, Memorial Sloan Kettering Cancer Center.

The combined probe has been explored primarily for the diagnosis and management of BCC (Figure 3A). In dermoscopically featureless, small (≤1 cm), equivocal lesions, Monnier et al. demonstrated a 100% sensitivity and 100% specificity for detecting BCC, which is both superior to RCM alone (90% sensitivity, 62.5% specificity) and OCT alone (90% sensitivity, 50% specificity) [63]. Sahu et al. observed a correlation between histopathological depth and OCT-estimated depth, with a coefficient of determination (R2) of 0.75 (R = 0.86; *p* < 0.001) [64]. They conclude that this depth assessment can aid in the selection of a treatment modality for BCC (i.e., surgical vs. non-surgical). In addition to the diagnosis of BCC, the use of RCM-OCT has been shown to be more effective in detecting residual BCC and delineating tumor margins than stand-alone RCM or OCT devices [61,65,66]. Aleissa et al. reported a sensitivity of 82.6% and a specificity of 93.8% for the detection of residual BCC in the surgical margin [67]. RCM-OCT has also served as an aid in the management of complex BCCs [66]. The use of a combined RCM-OCT probe may help by guiding treatment selection and defining the extent of surgery for BCCs.

Beyond its utility for BCC detection, this device has been explored for the diagnosis of other tumor types. Bang et al. showed potential for improving the detection of cutaneous metastases and differentiating them from vascular ectasia [68]. The authors described how RCM could detect tumor foci in the superficial dermis at a cellular resolution, while OCT aided in the detection of these foci in the deeper dermis. As cutaneous metastases also mimic primary cutaneous cancers, in future, this device could be also used to differentiate between these two entities. 

#### 3.2.2. High-Resolution Full-Field (FF)-OCT Devices 

The recently developed FF-OCT device (Supplemental Figure S1C) uses a Gaussian-like broadband light source, which eliminates the artifact known as ‘ghost image’ and can provide ultra-high cellular resolution in B-scan mode (vertical mode), similar to conventional histopathological tissue sections. FF-OCT yields cross-sectional images with an axial resolution of 1.35 μm, lateral resolution of 1.3 μm, and scanning depth of 400 μm. Compared with the RCM device, the FOV for FF-OCT is smaller (~500 × 400 μm) [69]. Using FF-OCT, Wang et al. described the features of various neoplasms, including actinic keratosis, Bowen’s disease, BCC, extramammary Paget’s disease, seborrheic keratosis, and large cell acanthoma [70,71]. The team further evaluated the feasibility of FF-OCT for the diagnosis and subtyping of BCCs (Figure 3B). They reported that even a reader who was inexperienced (defined as a single 13 min training in FF-OCT) can use this technique to diagnose BCC with a sensitivity of 75% and a specificity of 57%, and subtype them with a sensitivity of 50% and a specificity of 57% [72].

#### 3.2.3. Line Field (LC)-OCT

LC-OCT integrates the principle of OCT interferometry with the spatial filtering capabilities of RCM. This device provides high-resolution B-scan images in real time, with an isotropic spatial resolution of ∼1 μm up to a depth of ∼500 μm [73]. Compared with OCT, LC-OCT has a lower depth of penetration but superior resolution. Unlike RCM, which can provide only horizontal images, LC-OCT generates both vertical and horizontal images at a similar cellular resolution as RCM [74]. 

Features of both non-melanocytic and melanocytic skin cancers have been defined using the LC-OCT device [75,76,77,78]. Compared with RCM, LC-OCT has been shown to yield a higher diagnostic accuracy for the detection of BCC. Ruini et al. demonstrated a 90.4% (95% CI, 79.0–96.8) agreement of LC-OCT with conventional histopathology in diagnosing BCC subtypes, which is superior to OCT (84%) and RCM (62.5%) [76]. The same authors also showed that the LC-OCT can improve dermatologists’ confidence by 24.7% compared with clinical examination and dermoscopy [77] for the diagnosis of keratinocyte neoplasms (actinic keratosis, Bowen’s disease, and invasive SCC). LC-OCT has been used non-invasively to predict the progression of an actinic keratosis to an invasive SCC, using a proliferation (PRO) grading system. This grading on LC-OCT had a 75% agreement with the grading on histopathology [79].

This device has also shown a higher accuracy for differentiating nevi from melanoma. Schuh et al. demonstrated that the use of LC-OCT yields superior diagnostic accuracy (97% overall accuracy, 93% sensitivity, 100% specificity) as compared with the use of RCM (94% overall accuracy, 93% sensitivity, 95% specificity) [75]. However, large-scale studies are required to validate these results.

#### 3.2.4. Combined Multiphoton Microscopy (MPM)-RCM Device

Unlike RCM, which relies on the refractive index of various tissues, MPM is based on the nearly simultaneous absorption of two or more deeply penetrating near-infrared photons by endogenous fluorophores (keratin, melanin, etc.). Thus, it can be used to differentiate between various tissue structures. A combination of MPM with RCM enhances the capability to identify granules and minute particles of melanin and helps differentiate them from other morphologically similar fluorophores. Majdzadeh et al. accomplished the visualization of these melanin granules (both intracellular and extracellular) in the dermis and epidermis in a non-invasive manner even within non-lesional skin [80]. The utility of MPM-RCM in the detection of skin cancer remains understudied and warrants further exploration. 

In addition to cell morphology, MPM can be used to quantify collagen patterns, especially collagen type 1, and can aid in the diagnosis and prognostication of cancer. Sendín et al. recently demonstrated that, solely based on the second harmonic signal obtained from collagen type 1, BCC can be distinguished from healthy skin. They could further subtype BCC as nonaggressive or aggressive [81]. The collagen-based information could be integrated with cellular details on MPM and RCM to improve the diagnosis and management of BCC.

### 3.3. Addition of Fluorescent Targeted Molecular Probes to Improve Diagnostic Accuracy of RCM 

RCM imaging relies on the detection of singly backscattered light from sub-cellular structures. Melanin has the highest refractive index and appears bright, however, the nucleus has a weak backscatter from chromatin and appears dark [82]. This poses a major limitation in the visualization of tumors with a high nuclear-cytoplasmic ratio and increased nuclear density, such as BCC. To enhance the visualization of the nucleus, one may utilize exogenous molecule-targeted fluorescence nuclear contrast agents (e.g., poly (adenosine diphosphate-ribose), polymerase inhibitor-conjugated BODIPY-FL (PARPi-FL)) in combination with fluorescence (F)CM imaging. PARPi-FL has been shown to be overexpressed in BCC as compared with the surrounding normal adnexal structures (sebaceous gland, epidermis basal layer and hair follicle) and thus has the ability to provide a differential contrast. The use of PARPi-FL-labeled FCM imaging has the capability to improve the accuracy of diagnosis for BCC as compared with the use of reflectance (RCM) contrast alone; Sahu et al. demonstrated an improvement in sensitivity from 78.6–90% to 100% with slight or no improvement in specificity from 53.9% to 61.5% [83]. PARPi-FL dye is a small molecule that can penetrate intact skin via passive diffusion within 10–20 min to reach the dermis and label BCC tumor nodules; as such, it has acquired the status of a new investigational drug for use in head and neck cancer. Thus, PARPi-FL has a promising potential for use during in vivo imaging of patients [83].

### 3.4. Enhancement of Tumor Detection via EVCM with Fluorescent-Labeled Antibodies

Although acridine orange, a nuclear dye, enhances the contrast between the nucleus and dermis, it is not a tumor-specific dye. Thus, tumor-specific fluorescent-labeled antibodies (such as S-100, Melan-A, and Ber-EP4) have been explored for the intraoperative diagnosis of skin tumors. Hartmann et al. reported the detection of S100 signal and Melan-A signal with EVCM imaging in 83.3% and 63.9% of metastatic melanoma tissue, while a Ber-EP4-positive fluorescent signal was detected in 83.30% of BCC tissues [84].

### 3.5. Dynamic Observation of Tumor Microenvironment (TME) to Predict the Response to Immunotherapy

TME comprises micro-vasculature, inflammatory cells, and mucin-surrounding tumors. TME is known to influence anti-tumor immunity and the response to immunotherapy treatment [85]. A change in TME can be used to predict the immunotherapy response; such changes can be evaluated on conventional histopathology tissue sections [86]. However, histopathology requires a biopsy, which is a terminal phenomenon and cannot be used to monitor lesions during a treatment course. Furthermore, only static images can be evaluated at a single site, which may result in the suboptimal prediction of the treatment response. Sahu et al. used in vivo RCM to observe dynamic TME features (tumor angiogenesis and leukocyte trafficking) to predict the response to immunotherapy. The investigators showed a correlation of TME features seen in melanoma and BCC on RCM as compared with the gold standard of histopathology. The authors also reported that the treatment response to imiquimod therapy in patients with BCC can be predicted by features of TME within RCM phenotypes (intra-tumoral inflammation, number of vessels or tumor-infiltrating lymphocytes, number of vessels) with 71% sensitivity and 83% specificity [87].

### 3.6. Building More Affordable and Portable Microscopes for Widespread Use 

The currently available commercial CM devices (RCM and EVCM) are expensive, thus limiting their use in select large academic centers and in private clinics. Additionally, these devices are bulky and may not be practical for remote locations. In order to make CM devices more widely available across the world, manufacturers are producing more affordable and portable (smaller) versions of these devices [88,89]. For instance, to reduce the cost and complexity of conventional bulky RCM devices, a line-scanner RCM device has been developed. This device uses a single scanner and a linear-array detector that could drastically reduce the cost of this device to ~USD 15,000 [90]. 

A major innovation has been the development of a smartphone-attached-handheld-confocal microscope. These devices can visualize key cellular features of human skin in vivo with a comparable resolution to the commercially available confocal devices [91]. These portable devices are also inexpensive, as they use light-emitting diode (LED) as their light source and have an imaging sensor for capturing confocal images, instead of expensive lasers and bulky optoelectrical components (e.g., high-speed beam scanners, a fast data acquisition unit) used in the existing RCM devices [88,92]. This smartphone-based confocal microscope has been shown to have feasible use for the diagnosis of Kaposi’s sarcoma in resource-limited settings in Uganda [89]. Despite their inexpensive nature and small footprint, these devices have some limitations. These include difficulty in maintaining stable contact between the device and skin, which may result in blurry images and a low signal-to-noise ratio (SNR) [88]. 

Furthermore, the commercial handheld RCM devices have been transformed into telescopic devices that can be used for imaging intra-oral lesions [93]. Peterson et al. used such a device to assess intra-operative margins of an oral SCC in awake patients. As this device has a small FOV image (0. 75 mm × 0.75 mm), the video-mosaicking approach (Section 3.1) built for the commercial handheld device could be deployed to enable the visualization of a larger area (~4 mm × 2 mm) [94].

### 3.7. Integration of Artificial Intelligence (AI) Algorithms to Aid Novices with Confocal Microscopy Image Interpretation and Diagnosis

In addition to cost, another major limitation for the widespread use of these devices has been the inability of users to read confocal images. In vivo RCM images appear grayscale and in en-face view, thus requiring intensive training for novice readers to make correct diagnoses [12]. Although EVCM images are similar to H&E-stained ones, they may still require a trained pathologist or a Mohs surgeon for interpretation. AI or machine-learning-based approaches are widely integrated into various imaging modalities for the automated detection of cancers (such as lung cancers and lymph node metastases), including positron emission tomography (PET)/computed tomography (CT) [95]. Along a similar line, AI algorithms have been developed for both RCM and EVCM devices to overcome the limitations of image interpretation and aid novices in diagnosis [96,97,98].

#### 3.7.1. AI for Diagnosis and Interpretation of In Vivo RCM Images

Wodzinski et al. developed an artificial neural network (ResNet) and demonstrated its accuracy to be 87% in classifying common skin neoplasms (melanoma, BCC, and nevi) using in vivo RCM images, an accuracy that was slightly better than human readers’ ability [98]. Later, Campenella et al. developed a deep-learning-based AI model to automatically detect BCC in RCM images of clinically equivocal lesions. They showed that AI achieved a similar diagnostic accuracy as human expert readers with an area under the curve (AUC) for the receiver operator characteristic curve (ROC) of 89.7% (stack level) and 88.3% (lesion level). Their algorithm was also tested on an external dataset and demonstrated similar accuracy rates (AUC of 86.1%), indicating generalizability of the system’s performance [99]. Machine learning can assist the clinician with pattern recognition in pigmented lesions. Soenen et al. showed that the machine learning can increase the diagnostic accuracy in the differentiation of congenital pigmented macules, such as café au lait spots, from congenital nevi on RCM images [100]. Kose et al. developed an automated semantic segmentation method called Multiscale Encoder-Decoder Network (MED-Net). They showed that MED-Net could achieve a pixel-wise mean sensitivity and specificity of 70 ± 11% and 95 ± 2%, respectively, for the detection of various patterns of melanocytic lesions at the dermal/epidermal junction (DEJ) in in vivo RCM images. Moreover, MED-Net successfully identified the location and extent of the pattern with 0.71 ± 0.09 Dice coefficient [101].

To overcome the limitation of grayscale images, Li et al. used a convolutional neural network to convert grayscale in vivo RCM images into virtually stained H&E-like images. They described this approach to visualize various normal skin layers (epidermis, DEJ, and superficial dermis), BCC, and nevi in a virtually stained H&E-like mode [102]. Such an approach could improve the interpretation of the RCM images due to readers’ familiarity with the interpretation of H&E-stained images. 

In addition to the above diagnostic algorithms, AI models have been built to aid technicians in image acquisition and to improve the quality of in vivo RCM images. Several artifacts (corneal layer reflection, shifting, and misalignment of mosaics related to patient movement, relicts occurring due to convexity of nodular lesions, air and oil bubbles, skin creases) may arise during image acquisition, impacting the quality of in vivo RCM images. AI helps clinicians to minimize these artifacts by using deep neural networks and other approaches [103]. Kose et al. showed that MED-Net could automatically detect artifacts in RCM images with 82% sensitivity and 93% specificity [104]. 

AI algorithms have been also developed for portable confocal devices; their main purpose is to improve the SNR ratio, which is crucial for attaining cellular-level resolution images. Zhao et al. tested a content-aware image restoration (CARE) approach, which is one of the deep-learning-based computational methods, to denoise images in high-speed portable RCM to reduce SNR. With this approach, they achieved better noise reduction than the non-deep learning filtering methods yielded [105].

#### 3.7.2. AI for EVCM Image Diagnosis and Interpretation

Similar to their use in in vivo RCM images, AI algorithms have been built to facilitate the reading of the EVCM images. Sendín-Martín et al. developed a deep-learning algorithm that achieved a 92% diagnostic accuracy for the automatic detection of BCC in EVCM images [97]. Later, Combalia et al. showed that their AI approach (U-Net architecture) could also detect BCC in EVCM images, with a sensitivity of 88% and specificity of 91% [96]. On EVCM images, Ruini et al. reported a high potential of deep learning models to detect cutaneous SCCs and to distinguish them from tumor-free skin, with an overall sensitivity of 76% and specificity of 91% [106].

Certain technical issues can impact the image quality of the EVCM images. One of the biggest issues is flattening the tissue on the glass slide during imaging, which leads to an incomplete visualization of the epidermis. This may impact the diagnosis of epidermal tumors such as superficial BCCs and SCCs. Sendín-Martín et al. built a three-dimensional mosaicking and intensity projection to overcome these limitations [107]. Furthermore, Combalia et al. also developed an AI model (U-Net architecture) to achieve virtual tissue flattening. They also developed a coloring algorithm to improve the appearance of the EVCM images [96].

### 3.8. Remote Reading of Confocal Images

Interpreting RCM images necessitates extensive training, typically requiring a couple of years. Thus, the accuracy of diagnosis on RCM is related to the reader’s experience [12]. In order to integrate RCM imaging into clinical workflow, the novices need some assistance from an expert reader during imaging. As there is a paucity of expert readers worldwide, this limitation may be resolved via a telemedicine approach [50], which is now an integral part of dermatology and other medical fields. 

As RCM images are digital in nature, they can be read remotely. Remote interpretation can be achieved via two methods: a standard store-and-forward (SAF) method [108,109] and a new live interactive method (LIM) tele-RCM [110]. With the SAF method, images are transferred to a remote expert reader after they are acquired, while with the LIM tele-RCM, the expert joins the imaging session with access to the screen in real-time. The SAF method reportedly showed an improved diagnostic accuracy with the addition of a second (expert reader) opinion [109,111]. On the other hand, the LIM tele-RCM method has several advantages compared with the SAF method, including an interaction between the clinician and expert reader and the ability to train novice readers and guide technicians to acquire diagnostic images. Rubinstein et al. demonstrated the feasibility of the LIM tele-RCM approach for the detection of a BCC [110]. This method could also be useful during pandemics, such as COVID-19, for remote reading. Large-scale studies are ongoing to assess its diagnostic accuracy in cutaneous malignancies.

## Data Availability

Data sharing not applicable. No new data were created or analyzed in this study. Data sharing is not applicable to this article.

## Supplementary Materials

The following supporting information can be downloaded at: https://www.mdpi.com/article/10.3390/diagnostics13050854/s1, Figure S1: Photos of in vivo microscopes for used skin imaging; Table S1: Reflectance confocal microscopy (RCM) terms of the structures and their histopathological correlates in common skin malignancies.
