# Supplementary material for: Confocal Microscopy for Diagnosis and Management of Cutaneous Malignancies: Clinical Impacts and Innovation
Source: Diagnostics (Basel). 2023 Feb 23;13(5):854. doi: 10.3390/diagnostics13050854 (PMC10001140; doi:10.3390/diagnostics13050854)
Supplement: Supplementary file 1 [file diagnostics-13-00854-s001.zip › diagnostics-2158960-supplementary.pdf]

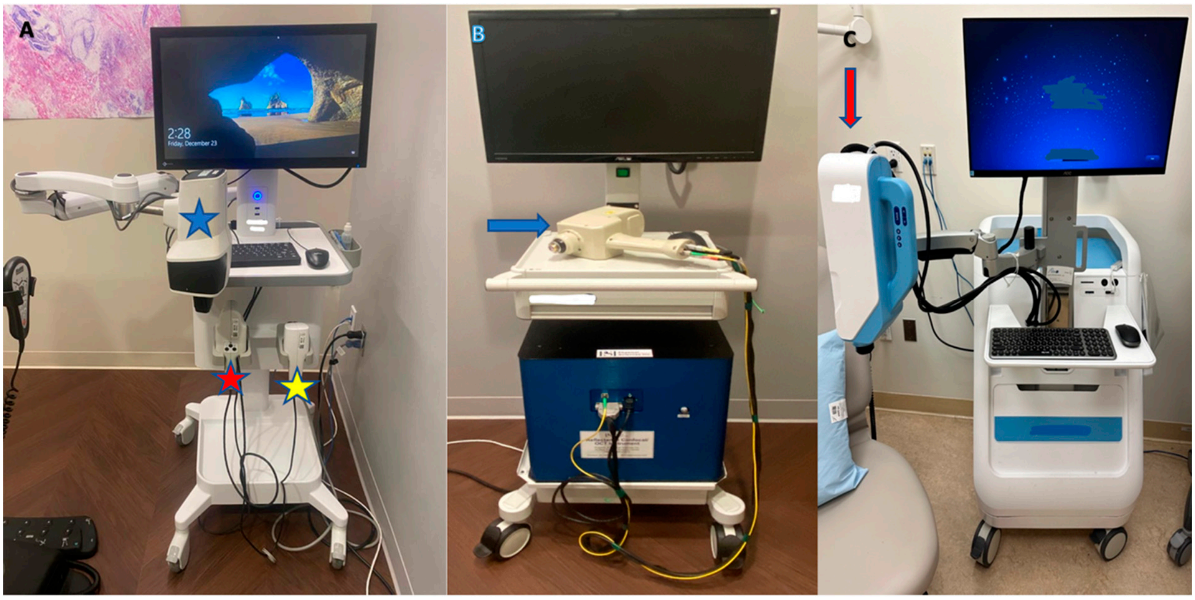

Supplemental Figure S1: Photos of in vivo microscopes for used skin imaging. (A) A reflectance confocal microscope mounted on a portable cart equipped with an arm mounted arm (blue star) and a handheld (red star) device and a dermatoscope (yellow star). (B) A combined RCM-OCT device with a handheld probe to acquire both OCT and RCM images, simultaneously (blue arrow). (C) A high-resolution Full-Field OCT (FF-OCT) microscope equipped an arm mounted arm piece for imaging (red arrow). Images courtesy: Ms. Ucalene Harris and Ms. Rozina Zeidan, MSKCC.

Supplementary Table S1: Reflectance confocal microscopy (RCM) terms of the structures and their histopathological correlates in common skin malignancies.

RCM Terms of the Structures	Histopathological Correlates
**Basal Cell Carcinoma (BCC)[a]**	
Streaming of the epidermis	Probably corresponds to the horizontal section of the uppermost part of a tumor nest or cord with palisading
Epidermal shadow	Probably an optical effect of the *en face* view of a tumor nest
Tumor island/nodules	Nests of basaloid tumor cells at the dermo-epidermal junction or in the superficial dermis
Peripheral palisading	Parallel arrangement of the basaloid tumor cells at the periphery of the tumor nests
Clefting	Space between the tumor parenchyma and the surrounding stroma
Cord-like structures/ Basaloid cords and nodules	Nests of tumor cells connected to the dermo-epidermal junction

Dark silhouettes	Nests of basaloid cells in the dermis, not clearly visible due to poor resolution at deeper depth (after 150–200 μm). Mostly seen in the infiltrative subtype of BCC
Increased vascularity	Increased number of dilated blood vessels running parallel to the epidermal surface
Peritumoral collagen reaction	Reaction of collagen to the dermal tumor nests
**Squamous Cell Carcinoma (SCC)**[a]	
Hyperkeratosis	Hyperkeratosis
Atypical honeycomb pattern	Alteration in nuclear and cellular size and shape of epidermal keratinocytes
Disarranged epidermal pattern	Severe alteration in nuclear and cellular size and shape of epidermal keratinocytes
Targetoid cells	Dyskeratotic keratinocytes within the epidermis
Keratin pearl	Keratinization
Buttonhole vessels (Round blood vessels traversing the dermal papilla)	Aberrant neovascularization
**Melanoma**[b]	
Pagetoid cells	Presence of atypical melanocytes in supra-basal layers of the epidermis
Polymorphic melanocytic cells (Bright dendrites/Dendritic/Round cells within the epidermis)	Presence of melanocytes in supra-basal layers of the epidermis.
Follicular localization of pagetoid cells and/or atypical cells	Dendritic projections of Langerhans cells or melanocytes atypical melanocytes in adnexal structures. Typical of lentigo maligna-type melanoma
Non-edged papillae	Disfiguration of the rete ridges due to atypical melanocyte proliferation out of the sides and the tip of rete ridges
Atypical cells at the dermo-epidermal junction	Proliferation of atypical melanocytes as an individual unit along the dermo-epidermal junction
Junctional nests/ Junctional thickening	Junctional nested proliferation of melanocytes
Sheet-like structures	Florid lentiginous proliferation of atypical melanocytes along the dermo-epidermal junction
Dense and sparse nests	Irregular or discohesive nests of melanocytes
Cerebriform nest	Nodular aggregates of atypical melanocytes in melanoma with dermal component
Plump cells	Melanophages

Patterns seen at lower-power magnification	
Ringed pattern	Lentiginous or small-nested junctional proliferation of melanocytes
Meshwork pattern	Nested junctional proliferation of melanocytes with bridging between adjacent rete ridges
Clod pattern	Dermal nested proliferation of melanocytes

Notes: [a] Navarrete-Dechent C, DeRosa AP, Longo C, Liopyris K, Oliviero M, Rabinovitz H, Marghoob AA, Halpern AC, Pellacani G, Scope A, Jain M. Reflectance confocal microscopy terminology glossary for nonmelanocytic skin lesions: A systematic review. J Am Acad Dermatol. 2019 May;80(5):1414-1427.e3. doi: 10.1016/j.jaad.2018.12.007. Epub 2018 Dec 8. PMID: 30529706; PMCID: PMC6830574. Also, this refers to the reference (25) in main text.

[b] Navarrete-Dechent C, Liopyris K, Monnier J, Aleissa S, Boyce LM, Longo C, Oliviero M, Rabinovitz H, Marghoob AA, Halpern AC, Pellacani G, Scope A, Jain M. Reflectance confocal microscopy terminology glossary for melanocytic skin lesions: A systematic review. J Am Acad Dermatol. 2021 Jan;84(1):102-119. doi: 10.1016/j.jaad.2020.05.097. Epub 2020 May 23. PMID: 32454102; PMCID: PMC8387955. Also, this refers to the reference (24) in main text.
